# Using low-risk factors to generate non-integrated human induced pluripotent stem cells from urine-derived cells

**DOI:** 10.1186/s13287-017-0698-8

**Published:** 2017-11-02

**Authors:** Linli Wang, Yuehua Chen, Chunyan Guan, Zhiju Zhao, Qiang Li, Jianguo Yang, Jian Mo, Bin Wang, Wei Wu, Xiaohui Yang, Libing Song, Jun Li

**Affiliations:** 1 Guangzhou Biocare Institute of Cancer, Building D, Guangzhou International Business Incubator, No. 3, Juquan Road, Guangzhou Science Park, Guangzhou, 510663 Guangdong People’s Republic of China; 2 The Guangdong Key Lab for Shock and Microcirculation Research, Departments of Pathophysiology, Southern Medical University, Guangzhou, 510515 People’s Republic of China; 3 State Key Laboratory of Oncology in Southern China and Department of Experimental Research, Sun Yat-sen University Cancer Centre, Guangzhou, 510060 People’s Republic of China; 4 Department of Biochemistry, Zhongshan School of Medicine, Sun Yat-sen University, 74 Zhongshan Road II, Yuexiu District, Guangzhou, Guangdong 510080 China

**Keywords:** Induced pluripotent stem cells, Human urinary cells, 6F/BM1-4C system, iPSC safety

## Abstract

**Background:**

Because the lack of an induced pluripotent stem cell (iPSC) induction system with optimal safety and efficiency limits the application of these cells, development of such a system is important.

**Methods:**

To create such an induction system, we screened a variety of reprogrammed plasmid combinations and multiple compounds and then verified the system’s feasibility using urine cells from different individuals. We also compared large-scale iPSC chromosomal variations and expression of genes associated with genomic stability between this system and the traditional episomal system using karyotype and quantitative reverse transcription polymerase chain reaction analyses.

**Results:**

We developed a high-efficiency episomal system, the 6F/BM1-4C system, lacking tumorigenic factors for human urine-derived cell (hUC) reprogramming. This system includes six low-risk factors (6F), *Oct4*, *Glis1*, *Klf4*, *Sox2*, *L-Myc*, and the miR-302 cluster. Transfected hUCs were treated with four compounds (4C), inhibitor of lysine-demethylase1, methyl ethyl ketone, glycogen synthase kinase 3 beta, and histone deacetylase, within a short time period. Comparative analysis revealed significantly decreased chromosomal variation in iPSCs and significantly increased *Sirt1* expression compared with iPSCs induced using the traditional episomal system.

**Conclusion:**

The 6F/BM1-4C system effectively induces reprogramming of urine cells in samples obtained from different individuals. iPSCs induced using the 6F/BM1-4C system are more stable at the cytogenetic level and have potential value for clinical application.

**Electronic supplementary material:**

The online version of this article (doi:10.1186/s13287-017-0698-8) contains supplementary material, which is available to authorized users.

## Background

Advancements in induced pluripotent stem cell (iPSC) technology have provided great opportunities for regenerative medicine and tumor immunotherapy [1–3]. However, human iPSCs (hiPSCs) are primarily induced using retroviral or lentiviral vectors carrying reprogramming factors [4, 5], and exogenous DNA fragments can randomly insert into genomic DNA and induce cell transformation, thereby preventing the clinical application of iPSCs. To date, many non-integrating methods have been generated, including mRNA and protein transfection [6, 7], Sendai virus (SeV) [8], piggyback (PB) transposons [9], and episomal vectors [10]. Nonetheless, mRNA and protein transfections are associated with high preparation costs and low induction efficiency, and a risk of transformation is associated with the retention of SeV RNA in the first passages of iPSC lines [8, 11] and PB transposons. Although episomal induction systems can avoid these problems and are widely used for reprogramming, most of these systems utilize at least one tumorigenic factor such as *c-Myc*, *SV40-LT*, and p53 inhibitors, including *p53* RNA interference (RNAi) or small molecule inhibitors [10, 12–17]. As induction efficiency varies when the same method is used to reprogram different types of somatic cells or when different methods are applied to reprogram the same type of somatic cells [12], it is crucial to optimize the induction method for each type of cell. Human urine-derived cells (hUCs) are ideal donors for iPSC generation: their isolation is simple and nontraumatic. In addition, these cells are easy to expand in vitro and can be used as the main source for iPSCs; thus, their use is cost-effective and universal [18–20]. In the present study, an episomal vector was used for hUC reprogramming [14, 17].

The nontransformative *MYC* family protein *L-Myc* can be replaced with *c-Myc* to induce iPSCs [15, 21]. *Glis1*, which is enriched in oocytes, can also replace *c-Myc* in the classical OSKM (*Oct4*, *Sox2*, *Klf4*, and *c-Myc*) system; chimeric mice generated from iPSCs induced using OSK and *Glis1* have longer survival times than those generated from iPSCs induced by OSKM [22]. Moreover, there is a positive correlation between chimeric mouse mortality and mouse tumor mortality [21], suggesting that *Glis1* is a safety factor for iPSC generation. The miR-302 family, which is specifically expressed in embryonic stem cells (ESCs), can partially or completely replace reprogramming factors and increase reprogramming efficiency [14, 23, 24]. Furthermore, the miR-302 family activates *Ink4a* and *Arf* to suppress the tumorigenesis of human pluripotency stem cells by targeting the oncogene *Bmi1* [25], and *Arf*/*p53* pathway activation suppresses somatic cell reprogramming [15, 16]. Therefore, miR-302 s are typically important factors that promote somatic cell reprogramming, but targeted factors that inhibit reprogramming exist in some signal pathways. Several studies to date have suggested that long noncoding RNAs (lncRNAs) regulate development and tumorigenesis; for example, long intergenic noncoding RNA, regulator of reprogramming (lincRNA-ROR) regulates the self-renewal and pluripotency of human ESCs (hESCs) and the reprogramming of hiPSCs [26, 27]. In this study, we applied hUCs as donor cells to induce iPSCs using low-risk factors, and then we screened a combination of low-risk reprogramming factors, including *Oct4*, *Glis1*, *Klf4*, *Sox2*, *L-Myc*, and the miR-302 cluster.

To improve non-integrated reprogramming efficiency, we optimized our culture system and observed iPSC induction with high efficiency when four compounds (Parnate, PD0325901, CHIR99021, and sodium butyrate) were added to the medium for no more than 4 days. To analyze iPSC safety, a karyotype analysis was performed, and the results showed that significantly lower iPSC chromosomal variation was induced when using this system than when using episomal systems containing *SV40-LT* and *c-Myc*.

## Methods

### Cell culture

hUCs were collected according to methods reported previously [14, 18]. Briefly, 100–1000 ml of urine was collected from donors, centrifuged at 1010 × *g* for 5 minutes, and washed with phosphate-buffered saline (PBS). The cells were maintained in 24-well plates coated with 0.1% gelatin (ES-006-B; Millipore, Germany) in RM1 medium (50% Renal Epithelial Cell Growth Medium (REGM) (CC-3190; Lonza, USA) and 44% Dulbecco's Modified Eagle Medium (DMEM) (SH30022; HyClone, USA) supplemented with 5% fetal bovine serum (FBS) (P30-3302; PAN Biotech, Germany), 0.5% nonessential amino acids (NEAA) (11140050; Gibco, USA), 0.5% GlutaMax (35050-061; Gibco, USA)) and 1 × Primocin (ant-pm-2; InvivoGen, USA); 0.25% trypsin-EDTA (25200072; Gibco, USA) was used for dissociation of primary hUCs. RM1 or RM2 (82% DMEM (SH30022; HyClone, USA) supplemented with 5% FBS, 1% human keratinocyte growth supplement (HKGS) (S-001-5; Gibco, USA), 1% NEAA, and 1% GlutaMax) was used for hUC culture.

The HN4 hESC line was obtained from the Chinese Academy of Sciences, and both HN4 and hiPSCs were maintained in the hESC medium BioCISO (BC-PM0001; BIOCARE Biotech, China) in plates coated with Matrigel (354277; Corning, USA).

### Plasmids

pCEP4 (V04450; Invitrogen, USA) was digested using the restriction enzymes *Nru*I and *Sal*I and ligated with synthesized multiple cloning site (MCS) oligonucleotides to obtain the plasmid pE2.1. The EF1α promoter (*Nru*I, *Nhe*I), BGH-PA element (*Bam*HI, *Pme*I), *EF1α* promoter (*Pme*I, *Bgl*II), and BGH-PA element (*Pac*I, *Sal*I) were cloned into pE2.1 to obtain the plasmid pE3.1. The process chart for pE3.1 plasmid construction is shown in Additional file 1: Figure S1a. To obtain the plasmid pE3.2, the *EF1α* promoter (*Pme* I, *Bgl* II) was replaced with the cytomegalovirus (CMV) promoter (*Pme*I, *Bgl*II). The sequences of *Oct4*, *Sox2*, and *Klf4* were subcloned from OKSIM (Plasmid 24603; Addgene, USA). The *Glis1* sequence was subcloned from pMXs-Glis1 (Plasmid 30166; Addgene, USA) and the *L-Myc* sequence from pMXs-Hu-L-Myc (Plasmid 30166; Addgene, USA). The Oct4-P2A-Glis1, Klf4-P2A-Sox2, and Oct4-P2A-L-Myc sequences were obtained using overlap PCR. The sequence of the miR-302 cluster was cloned from genomic DNA. The exon sequences of lincRNA-ROR were amplified using genomic DNA and synthesized DNA, and overlap PCR was used to obtain the complete lincRNA-ROR sequence. These DNA sequences were cloned into the plasmids pE3.1 and pE3.2, respectively, to generate the plasmids pE3.1-OL--KS, pE3.1-OG--KS, pE3.1-Oct4--Klf4, pE3.1-Glis1--LINC-ROR, pE3.1-L-Myc--hmiR-302 cluster, pE3.1-Oct4--Sox2, and pE3.2-L-Myc--hmiR-302 cluster. Information regarding the factors, primer sequences, and MCS is shown in Additional file 2: Table S1.

### iPSC generation

For hUC16 reprogramming, 1.5 × 10^6^ hUC16 cells were transfected with plasmids (Additional file 3: Table S2) using the T-020 program of a Lonza Nucleofector 2b Device and a Basic Epithelial Cells Nucleofector Kit (VPI-1005; Lonza, USA). Transfected hUC16 cells were seeded into six-well plates coated with 0.1% gelatin and cultured using RM2 medium. After 24 h, the cells were dissociated using 0.25% trypsin-EDTA (25200-072; Gibco, USA), and 2 × 10^4^ cells were seeded into 12-well plates coated with Matrigel. To induce iPSCs, the medium was changed to BioCISO-BM1 medium (BC-BM001; BIOCARE Biotech, China) containing 4i (A83-01 (0.5 μM, BC-SMC-A01-10; BIOCARE Biotech, China), Thiazovivin (0.5 μM, BC-SMC-T01-10; BIOCARE Biotech, China), CHIR99021 (3 μM, BC-SMC-C01-10; BIOCARE Biotech, China), and PD03254901 (0.5 μM, BC-SMC-P01-10; BIOCARE Biotech, China)) after 24 h. The medium was then changed to BioCISO on day 15. Alkaline phosphatase (AP) staining was performed on day 18, and the induction efficiency was calculated according to the formula:

$$ \mathrm{Induction}\  \mathrm{efficiency}=\mathrm{AP}\hbox{-} \mathrm{positive}\  \mathrm{colony}\  \mathrm{number}/\mathrm{total}\  \mathrm{seeded}\  \mathrm{cell}\  \mathrm{number}\times 100\%. $$

For reprogramming using the 6F/BM1-4C system, approximately 2.8 × 10^6^–3.5 × 10^6^ hUCs were transfected with 4.0 μg pE3.1-OG--KS and 2.8 μg pE3.1-L-Myc--hmiR-302 cluster using the same nucleofector method. The transfected hUCs were placed in plates coated with Matrigel and cultured with RM1 medium. On day 3 after nucleofector addition, the medium was changed to BioCISO-BM1 medium containing 2 μM Parnate (also known as tranylcypromine hydrochloride, 1986-47-6; Curegenix, China). The medium was then changed to BioCISO-BM1 medium containing 2 μM Parnate, 0.25 mM sodium butyrate (NaB) (303410-100G; Sigma, USA), 3 μM CHIR99021, and 0.5 μM PD03254901 on day 5, to BioCISO-BM1 on day 7, and to BioCISO on day 17. iPSC colonies were collected or stained with AP on day 19. The induction efficiency was calculated according to the formula:

$$ \mathrm{Induction}\  \mathrm{efficiency}=\mathrm{AP}\hbox{-} \mathrm{positive}\  \mathrm{colony}\  \mathrm{number}/\left(\mathrm{nucleofector}\  \mathrm{cell}\  \mathrm{number}\hbox{--} \mathrm{death}\  \mathrm{cell}\  \mathrm{number}\right)\times 100\%. $$

Compounds used in the present study also included dimethyloxaloylglycine (DMOG) (0.1 μM, D1070; Frontier Scientific, USA), PS48 (5 μM, 1180676-32-7; Curegenix, China), SC-79 (0.5 μM, 4635; Tocris, USA), forskolin (5 μM, 66575-29-9; Curegenix, China), and 3-deazaneplanocin A (DZNEP) (0.05 μM, 4703; Tocris, USA).

For reprogramming using the 4F2L-6C system, 3.0 × 10^6^ hUCs were transfected with 4.0 μg pEP4-E02S-ET2K and 2.8 μg pCEP4-M2L using the same nucleofector method. The transfected hUCs were placed in plates coated with Matrigel and cultured with RM1 medium. The medium was changed to BioCISO-BM1 medium containing 2 μM Parnate on day 3 after nucleofector addition, and then changed to BioCISO-BM1 medium containing 2 μM Parnate, 0.25 mM NaB, 3 μM CHIR99021, 0.5 μM PD03254901, 0.5 μM A83-01, and 0.5 μM Thiazovivin on day 5. The medium was changed to BioCISO-BM1 on day 7 and to BioCISO on day 17. iPSC colonies were collected on day 34.

### iPSC characterization

AP staining, non-integrated PCR analysis, flow cytometry analysis, immunofluorescence analysis, bisulfate sequencing, in-vitro embryoid body (EB) differentiation assays, and in-vivo teratoma formation were conducted according to previous methods [14, 17]. Briefly, AP staining was carried out using nitroblue tetrazolium (NBT) (N104908-1 g; Aladdin, China) and 5-bromo-4-chloro-3-indolyl phosphate (BCIP) (BIMB1018; J&K Chemical Technology, China). PCR was applied to analyze the integration of exogenous reprogramming factors and the episomal backbone with the primers presented in Additional file 2: Table S1. Flow cytometry and immunofluorescence analyses were employed to examine human pluripotency markers with the following antibodies: anti-Oct4 (130-105-606; Miltenyi Biotec, Germany), anti-SSEA4 (sc-21704; Santa Cruz, USA), anti-Tra-1-60 (sc-21705; Santa Cruz, USA), anti-Tra-1-81 (sc-21706; Santa Cruz, USA), anti-IgM-PE (sc-3768; Santa Cruz, USA), and anti-IgG3-PE (sc-3767; Santa Cruz, USA). Bisulfate sequencing was used to determine *Oct4* and *Nanog* promoter methylation with the primers presented in Additional file 2: Table S1. The PCR product was cloned into the pMD18-T vector and subsequently sequenced. For the in-vitro EB differentiation assay, cells were scraped from plates after dissociation using BioC-PDE1 (BC-PDE1; BIOCARE Biotech, China) and cultured in six-well suspension culture plates (657185; Greiner, Germany) with BioCISO-EB1 medium (BC-EB001; BIOCARE Biotech, China) for 7 days to obtain EBs. The EBs were then cultured in six-well culture plates (657160; Greiner, Germany) coated with Matrigel for 7–14 days. The cells were collected, and expression profiles of marker genes in the three germ layers were determined using quantitative reverse transcription PCR (qRT-PCR) with primers obtained from BIOCARE Biotech (BioEB-primer). For in-vivo teratoma formation, iPSCs were cultured to approximately 85% confluence; after 10–15 minutes of dissociation using BioC-PDE1, cells were scraped from the plates. The hind-limb muscle and forelimb subcutaneous muscle of 6-week-old NOD/SCID mice were injected with 130 μl BioCISO culture medium, 70 μl Matrigel, and cell suspensions. The formation of teratomas could be observed after 6–8 weeks; when the teratomas reached a certain size, they were removed and fixed with 4% paraformaldehyde. The tissues were embedded with paraffin, sectioned, stained with hematoxylin and eosin, and analyzed under a microscope. The procedures were performed according to IACUC (Institutional Animal Care and Use Committee; YS-YFStudy060-20160315).

### Karyotype analysis

Sample preparation for karyotyping was conducted as described previously [28]. Cells were treated with 50 ng/ml colchicine (Xy008; Xiangya Gene Technology, China) for 16 h, and the Ikaros karyotyping system was used to analyze karyotypes. The aneuploid evaluation is shown in Additional file 4: Table S3.

### Quantitative reverse transcription polymerase chain reaction

Total RNA was isolated using RNAiso Plus (TaKaRa), and M-MLV Reverse Transcriptase (TaKaRa) was used to synthetize cDNA. Specific stem-loop primers and random primers were used for reverse transcription of microRNAs and mRNAs into cDNA, respectively. mRNA and miRNA expression levels were determined using SYBR Premix Ex Taq™ (TaKaRa). Reactions were performed in triplicate using a LightCycler 480II/96 system (Roche, Switzerland). mRNA expression was normalized to GAPDH, and microRNA (miRNA) expression was normalized to U6 small nuclear RNA (snRNA). The primers are presented in Additional file 2: Table S1.

### Western blot analysis

Radioimmunoprecipitation assay (RIPA) buffer (CW2333; Cwbiotech, China) supplemented with a protease inhibitor cocktail (PI003; BOCAI Technology, China) and phenylmethylsulfonyl fluoride (PMSF; Dingguo Changshen Biotech, China) was used to isolate cellular proteins. Equivalent amounts of protein were separated by sodium dodecyl sulfate polyacrylamide gel electrophoresis (SDS-PAGE) and transferred to polyvinylidene difluoride (PVDF) membranes. The membranes were incubated with specific primary antibodies against Oct4 (2840; Cell Signaling Technology, USA), Glis1 (SAB2700289; Sigma, USA), Klf4 (ab72543; Abcam, UK), Sox2 (3579; Cell Signaling Technology, USA), L-Myc (sc-790; Santa Cruz Biotechnology, USA), and GAPDH (KC-5G4; KangChen Biotech, China), followed by horseradish peroxidase-conjugated secondary antibodies: goat anti-Rabbit IgG (ZB-2301; ZsBio, China) and anti-mouse IgG-HRP (IH-0031; Dingguo Changshen Biotech, China). Bands were visualized using enhanced chemiluminescence (ECL) (34087; Thermo, USA).

### Microarray analysis

GeneChip Human Transcriptome Array 2.0 (Affymetrix HTA 2.0, USA) was utilized to determine the gene expression profiles of human ESCs, iPSCs, and hUCs. The experiments were conducted according to the manufacturer’s instructions.

### Statistical analysis

SPSS 18.0 was used to perform statistical analysis. The results are presented as the mean ± standard deviation (SD) of at least three repeated individual experiments for each group. Statistical differences were examined using Student’s *t* test. For analysis of the chromosome abnormality rate, a four-table chi-square test was applied. *P* < 0.05 was considered statistically significant.

### Accession numbers

Microarray data for human ESs, iPSCs, and hUCs have been submitted to Gene Expression Omnibus (http://www.ncbi.nlm.nih.gov/geo/) under accession number GSE85885.

## Results

### Screening low-risk reprogramming factors using hUCs

Since Yamanaka used OSKM to induce reprogramming, many genes and non-RNAs that improve reprogramming efficiency have been reported. To induce reprogramming in this study, we employed low-risk factors, including *L-Myc*, *Glis1*, lincRNA-ROR, and the miR-302 cluster, and randomly combined them with *Oct4*, *Sox2*, and *Klf4* in the Epstein–Barr virus-encoded nuclear antigen-1 (EBNA)-oriP episomal vector (Additional file 1: Figure S1a, S1b, S1c). The miR-302 family can increase reprogramming efficiency by replacing reprogramming factors [14, 23, 24]. Furthermore, miR-302 family members target the oncogene *Bmi1* and suppress tumorigenesis, which can inhibit somatic cell reprogramming [15, 16, 25]. Therefore, miR-302 s have a positive impact on somatic cell reprogramming, but in some pathways these family members can inhibit reprogramming by indirectly activating targets that inhibit reprogramming. Because the expression levels of the miR-302 cluster must be precisely regulated, we used different promoters to exogenously express the miR-302 cluster and screened for optimal expression (Additional file 1: Figure S1a, S1b).

**Fig. 1:**
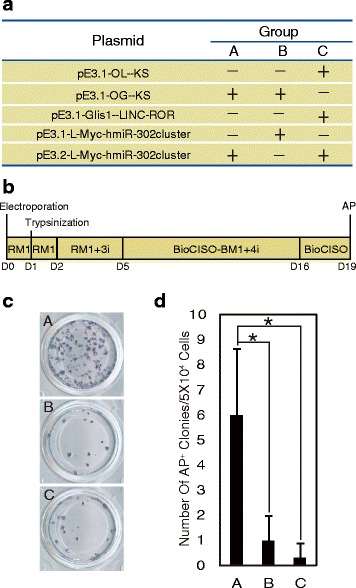
Screening low-risk factors for iPSC generation. **a** Screening strategy for the optimal plasmid combination. **b** Time schedule of iPSC generation. 3i = 0.5 μM A83-01, 3 μM CHIR99021, 0.5 μM thiazovivin. **c** AP staining to identify iPSCs. **d** Number of AP-positive colonies. Error bars indicate mean ± SD. *P < 0.05. *P*(B) = 0.038, *P*(C) = 0.022. AP alkaline phosphatase, D day

To evaluate the best plasmid combination for reprogramming, we used hUC16 cells constructed in our laboratory that showed high proliferation (Additional file 5: Figure S2a) and transfected these cells with different plasmid combinations (Additional file 5: Figure S2b), followed by AP staining to identify iPSCs after 18 days of nucleofection. Three groups of cells harboring the reprogramming factors *Oct4*, *Glis1*, *Klf4*, *Sox2*, *L-Myc*, lincRNA-ROR, and the miR-302 cluster with high AP-positive scores were selected for further analysis (Additional file 5: Figure S2c, S2d). The initial screen was performed in cells (UC16) with high proliferative ability and strong anti-stress capacity, and the best three combinations were further tested using other hUCs to ensure reliability. To induce reprogramming, we transfected the three plasmid combinations into hUCs and cultured in hESC basal medium (BioCISO-BM1) with the addition of four small inhibitors that have been widely used for reprogramming [12, 29]: 4i, the TGF-β/Activin/Nodal receptor inhibitor A83-01 (0.5 μM); the MEK inhibitor PD0325901 (0.5 μM); the GSK3β inhibitor CHIR99021 (3 μM); and the ROCK inhibitor Thiazovivin (0.5 μM) (Fig. 1a, b). As revealed by AP staining, the combination termed 6F, which includes *Oct4*, *Glis1*, *Klf4*, *Sox2*, *L-Myc*, and the miR-302 cluster initially expressed from the CMV promoter (Fig. 1c, d), showed the highest reprogramming efficiency at 19 days post nucleofection. Thus, we selected 6F, which does not contain high-risk tumorigenic factors such as *c-Myc*, *SV40-LT*, and p53 inhibitors, as the reprogramming induction combination for hUCs and found that it successfully induced hUCs into iPSCs.

### Optimization of compounds in the 6F combination system

**Fig. 2:**
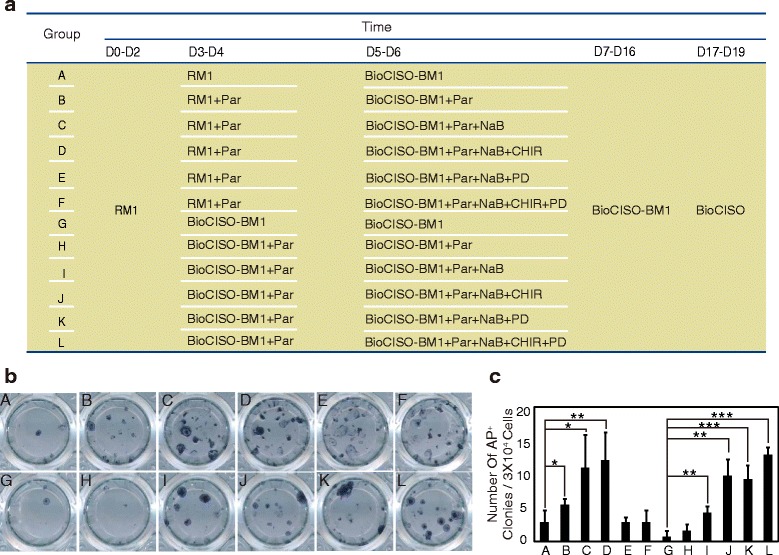
Optimization of the 6F combination system. **a** Screening strategy for six-factor combinations. **b** AP staining to evaluate iPSC generation. **c** Numbers of AP-positive iPSCs induced using different strategies. Error bars indicate mean ± SD. *P < 0.05, **P < 0.01, ***P < 0.001. *P*(B) = 0.038, *P*(C) = 0.022, *P*(D) = 0.006, *P*(I) = 0.002, *P*(J) = 0.001, *P*(K) = 0.000, *P*(L) = 0.000. AP alkaline phosphatase, D day, PD PD0325901, CHIR CHIR99021, NaB sodium butyrate, Par Parnate

Different cell lineages exhibit different gene expression profiles, and ideal reprogramming factor combinations and induction conditions depend on the cell type [12, 30, 31]. To determine whether 4i is the best induction condition for the 6F combination, we first examined the effects of compounds that are reported to regulate reprogramming in our system, including many compounds involved in signaling pathways. Some inhibitors or activators were observed to be unsuitable for our system; for example, the TGF-β/Activin/Nodal receptor inhibitor A83-01 and the ROCK inhibitor Thiazovivin did not promote programming (Additional file 6: Figure S3a–S3f). In addition, other compounds [31, 32], such as the PDK1 activator PS48, adenylyl cyclase activator forskolin, histone methyltransferase inhibitor DZNEP, HIF-α prolyl hydroxylase inhibitor DMOG, and Akt activator SC-79, did not promote or inhibit programming (Additional file 6: Figure S3g–S3l). Next, we examined the effects of combinations of compounds that promote reprogramming on the reprogramming efficiency. These compounds included the MEK inhibitor PD0325901 (PD, 0.5 μM), the GSK3β inhibitor CHIR99021 (CHIR, 3 μM), the histone deacetylase inhibitor NaB (0.25 mM), and the lysine-specific demethylase 1 inhibitor Parnate (Par, 2 μM). We observed that when Par was used in 6F combination reprogramming for an extended time interval, many cells in the cell layer shrank and were floating; however, the reprogramming efficiency increased when Par was added for a short time period (Additional file 6: Figure S3m–S3q). Thus, compound combinations, including Par, were used for no longer than 4 days. AP staining showed the highest reprogramming efficiency and the best iPSC quality when Par was added to the BioCISO-BM1 medium on days 3–4 and when PD, CHIR, NaB, and Par, termed 4C, were added on days 5–6 (Fig. 2a–c). Therefore, we selected BM1-4C as the optimum induction culture condition for the 6F combination induction system and named it 6F/BM1-4C.

### Reprogramming hUCs from different human sources using the 6F/BM1-4C system

**Fig. 3:**
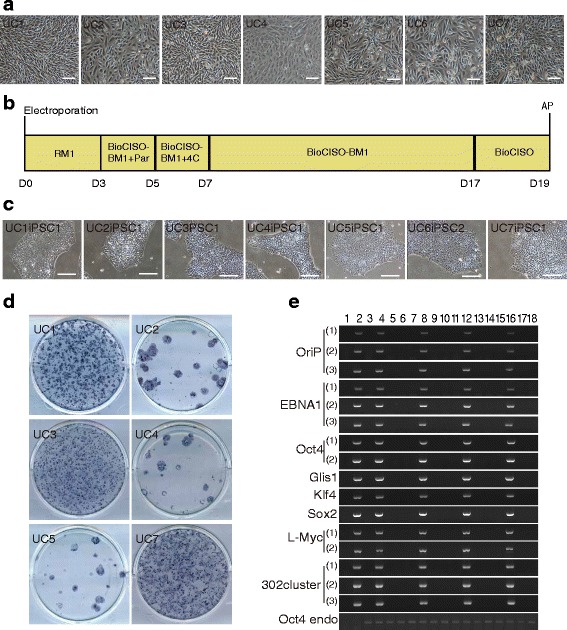
Induction of iPSCs from multiple hUCs using the 6F/BM1-4C system. **a** Morphology of hUCs isolated from seven different donors. **b** Time schedule of the 6F/BM1-4C reprogramming system. **c** Morphology of iPSCs induced from seven groups of hUCs using the 6F/BM1-4C system. **d** AP staining for iPSC generation from multiple hUCs using the 6F/BM1-4C system. **e** Non-integrating analysis of episomal DNA in iPSCs. Representative lanes: 1, H_2_O; 2, pE3.1-OG--KS and pE3.2-L-Myc--hmiR-302 cluster; 3, UC1; 4, UC1, pE3.1-OG--KS, and pE3.2-L-Myc--hmiR-302 cluster; 5, UC1iPSC1; 6, UC1iPSC2; 7, UC2; 8, UC2, pE3.1-OG--KS, and pE3.2-L-Myc--hmiR-302 cluster; 9, UC2iPSC1; 10, UC2iPSC2; 11, UC3; 12, UC3, pE3.1-OG--KS, and pE3.2-L-Myc--hmiR-302 cluster; 13, UC3iPSC1; 14, UC3iPSC2; 15, UC4; 16, UC4, pE3.1-OG--KS, and pE3.2-L-Myc--hmiR-302 cluster; 17, UC4iPSC1; 18, UC4iPSC2. Scale bars, 100 μm. AP alkaline phosphatase, D day, Par Parnate

**Fig. 4:**
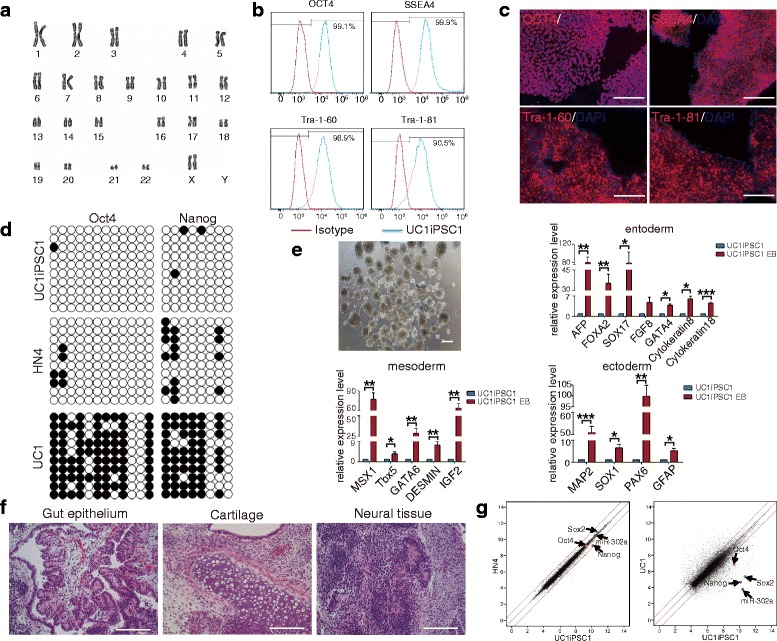
Pluripotent characterization of iPSCs induced from UC1 cells using the 6F/BM1-4C system. **a** Karyotype analysis of iPSCs induced from UC1 cells. **b** Flow cytometry assay for expression of the hESC markers OCT4, SSEA4, Tra-1-60, and Tra-1-81. **c** Immunofluorescence assay for expression of hESC markers. **d** Bisulfite sequencing assay for the methylation status of the *Oct4* and *Nanog* promoters in iPSCs. **e** In-vitro differentiation assay for UC1 iPSCs, and EB morphology. **f** Hematoxylin and eosin staining of sections of teratomas generated from UC1 iPSCs. **g** Scatter plots comparing global gene expression patterns between HN4 hESCs and UC1 iPSCs and between UC1 cells and UC1 iPSCs. Highlighted are the pluripotency factors Oct4, Sox2, Nanog, and miR-302a. Error bars indicate mean ± SD. *P < 0.05, **P < 0.01, ***P < 0.001. Scale bars, 100 μm. EB embryoid body

When hUCs were isolated from different individuals or from the same individual at different time points, the cells exhibited different morphologies when cultured, suggesting that these cells were of different types, which is consistent with previous reports [17, 33–35]. To confirm that the 6F/BM1-4C system is suitable for reprogramming different types of hUCs, we reprogrammed seven groups of hUCs isolated from different individuals (Fig. 3a, b, Table 1). At approximately 19 days post nucleofection, we collected iPSCs for further purification and expansion culture (Fig. 3c). The remaining iPSCs were subjected to AP staining, which demonstrated that the 6F/BM1-4C system resulted in effective reprogramming of all hUCs into iPSCs. AP staining also revealed an AP-positive rate that varied between 0.00021 and 0.0741% for iPSCs reprogrammed from different types of hUCs (Fig. 3d, Table 1). Furthermore, PCR analysis demonstrated a lack of genomic integration of the exogenous gene sequence in 13 of 14 iPSC colonies (Fig. 3e, Additional file 7: Figure S4a). Moreover, karyotype analysis revealed that 13 iPSC colonies had normal chromosome numbers and G-band distributions (Fig. 4a, Additional file 7: Figure S4b). Flow cytometry expression and immunofluorescence analyses revealed that iPSCs induced using the 6F/BM1-4C system express the hESC-specific markers Oct4, SSEA4, Tra-1-60, and Tra-1-81, suggesting that 6F/BM1-4C-iPSCs possess the molecular characteristics of hESCs (Fig. 4b, c, Additional file 8: Figure S5a, S5b). In addition, bisulfite sequencing PCR analysis indicated that the endogenous pluripotency genes *Oct4* and *Nanog* were activated and that their promoters were demethylated in 6F/BM1-4C-iPSCs, similar to what occurs in hESCs (Fig. 4d, Additional file 8: Figure S5c). The generated iPSCs were also able to differentiate into derivatives of all three germ layers, as determined using an in-vitro EB differentiation assay and an in-vivo teratoma formation assay (Fig. 4e, f, Additional file 8: Figure S5d, S5e). The gene expression profile of iPSCs was similar to that of hESCs and differed from that of hUCs, which was determined using Affymetrix gene microarray HTA 2.0. (Fig. 4g, Additional file 8: Figure S5f). The induction efficiencies of the different sources of hUCs as well as the iPSC characteristics are presented in Table 1. Together, these results indicate that the 6F/BM1-4C system has high reliability and versatility for reprogramming hUCs into iPSCs.

**Table 1:** Induction efficiency and characterization of iPSCs induced from hUCs using the 6F/BM1-4C system

Donor	Age	Gender	Cell number	Death rate^a^ (%)	Efficiency (%)	No.	Non-integration	Karyotype	FACS	BSP	Teratoma^b^	EB formation	Immunofluorescent
1	23	Female	3 × 10^6^	53.8	0.0468	UC1iPSC1	+^c^	+	+	+	+	+	+
						UC1iPSC2	+^c^	+	+	–	–	–	–
2	26	Male	2.8 × 10^6^	80.37	0.0015	UC2iPSC1	+^c^	+	+	–	+	+	+
						UC2iPSC2	+^c^	+		+	–	–	–
3	27	Male	3 × 10^6^	50	0.0741	UC3iPSC1	+^c^	+	–	–	+	+	–
						UC3iPSC2	+^c^	+	–	+	–	–	–
4	26	Female	3 × 10^6^	71	0.00089	UC4iPSC1	+^c^	+	–	–	–	–	–
						UC4iPSC2	+^c^	+	–	+^e^	–	–	–
5	24	Male	3 × 10^6^	66.35	0.00095	UC5iPSC1	+^c^	+	–	–	+	–	–
						UC5iPSC2	+^c^	+	–	+^e^	–	–	–
6	30	Female	2.53 × 10^6^	36	0.00021	UC6iPSC1	+^d^	+	–	–	–	–	–
						UC6iPSC2	+^c^	+	–	+	–	–	–
7	29	Male	3 × 10^6^	27.5	0.0429	UC7iPSC1	+^c^	+	–	+^e^	–	–	–
						UC7iPSC2	+^c^	+	–	–	–	–	–

At nucleofection, the hUC passage number was 2

+ iPSCs identified, – characterization not identified, *iPSC* induced pluripotent stem cell, *hUC* human urine-derived cell, *FACS* fluorescence-activated cell sorting, *EB* embryoid body, *HE* hematoxylin and eosin

^a^Taking supernatant (containing nonadherent cells) to count cells on the 3rd day after electronic transformation, the amount of counted cells was the amount of dead cells. Death rate calculated as: death rate = amount of dead cells/amount of cells for electroporation

^b^We injected five different donor-derived iPSCs in the teratoma experiment. Each NOD/SCID mouse was injected with a cell. In 6–8 weeks, three NOD/SCID mice formed teratomas and were dissected and HE stained. In the 8th week, one NOD/SCID mice had granule-shaped teratoma, and the teratoma was taken out and HE stained in the 12th week. One mouse did not form teratoma by observation

^c^Exogenous gene sequence did not integrate in the genome

^d^Exogenous gene sequence integrated in the genome

^e^Corresponding primary cells did not detect the pluripotency gene promoter DNA methylation due to the exhausted cells

### The 6F/BM1-4C system is safer than traditional episomal induction systems

**Fig. 5:**
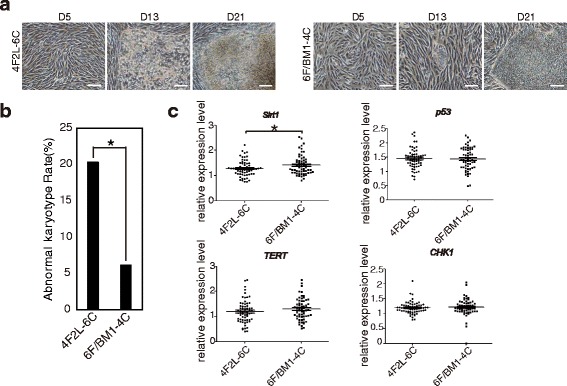
Safety comparison between 6F/BM1-4C and a traditional episomal induction system. **a** Changes in morphology in the iPSC generation process using the 4F2L-6C and 6F/BM1-4C systems. **b** Abnormal karyotype rates of iPSCs generated using the 4F2L-6C (*n* = 64) and 6F/BM1-4C (*n* = 65) systems. *P < 0.05 (*P* = 0.017). **c** Quantitative real-time PCR assay for *Sirt1*, *p53*, *TERT*, *CHK1* expression. *P < 0.05 (*P* = 0.034). Scale bars, 100 μm. D day

We observed that before day 5 following nucleofection, the observed cell masses had high nuclear–cytoplasmic ratios, a phenotype that was similar to the lentiviral preprogramming process. From days 9 to 11, the colony mass underwent massive death, and the surviving cells grew slowly to maturity. However, because early induction using the traditional episomal induction system (termed 4F2L-6C), which includes *Oct4*, *Sox2*, *Klf4*, *c-Myc*, *SV40-LT*, and *Lin28* [12], is a gradual process, the cells aggregated slowly and displayed little massive death (Fig. 5a, Additional file 9: Figure S6a, S6b); these results suggest that the two induction systems differ regarding iPSC generation. Therefore, we examined the safety of iPSCs generated using the 6F/BM1-4C system.

Evaluation of the application of iPSCs is based on safety. Schlaeger et al. [36] analyzed chromosomal variation among iPSCs induced using retrovirus, mRNA transfection, SeV, episomal, and lentivirus systems and observed large differences using these various methods. For example, the aneuploidy rate of iPSCs induced using the mRNA transfection method was only 2.3%, whereas that of iPSCs induced episomally was as high as 11.5%. However, these methods employ different reprogramming genes, and it is difficult to determine whether differences in chromosomal variation reflect the genes or the methods used for induction. In our study, we utilized an episomal vector to reprogram the same batch of hUCs isolated from the same donor using the 6F/BM1-4C and 4F2L-6C systems, and at least 60 iPSC colonies (65 for 6F/BM1-4C and 64 for 4F2L-6C) were selected for karyotype analysis (the specific criteria are summarized in Methods). We observed a significantly lower chromosomal abnormality rate for 6F/BM1-4C-iPSCs than for 4F2L-6C-iPSCs (*P* = 0.017, χ^2^ test; Fig. 5b, Table 2). We also determined the expression profiles of genes associated with genomic stability, such as *Sirt1*, *p53*, and *CHK1*, and found significantly high *Sirt1* expression in iPSCs induced using the 6F/BM1-4C system (Fig. 5c). These data show that iPSCs induced using the 6F/BM1-4C system are safer than those induced using the traditional episomal induction method, which would be beneficial for clinical applications.

**Table 2:** Specific changes in cell karyotypes using the 4F2L-6C and 6F/BM1-4C systems

No.	Method	Karyotype change(s)
7	4F2L-6C	88 < 4n>,XXXX,-X,-19,-18,-18[20]
13	4F2L-6C	46 < 2n>,XX,der(2)ins(2;?)(P25;?)[20], 89 < 4n>,XXXX,-XX,-5[20]
14	4F2L-6C	87 < 4n>,XXXX,-XX,-15,-19,-19[20]
18	4F2L-6C	89 < 4n>,XXXX,-17,-17,-15?
19	4F2L-6C	46 < 2n>,XX,der(13)t(13;1)(p13;q11 → q44)[20]
22	4F2L-6C	45 < 2n>,XX,-17[20]
29	4F2L-6C	91 < 4n>,XXXX,der(15)t(15;22)(p13;p13 → q13),-5[20]
37	4F2L-6C	46 < 2n>,XX,+9[20]
40	4F2L-6C	45 < 2n>,XX,-7[20]/-17[20]/-21[20], 47 < 2n>,XX,+15[20]
49	4F2L-6C	45 < 2n>,XX,-X[20], 92 < 4n>,XXXX,der(17)ins(17;?)(p13;?)
54	4F2L-6C	92 < 4n>,XXXX,-X,+11[20]
57	4F2L-6C	45 < 2n>,XX,-9[20]/-17[20]
60	4F2L-6C	92 < 4n>,XXXX,-X,+22[20]
3	6F/BM1-4C	90 < 4n>,XXXX,-16,-16, 45 < 2n>,XX,-22[20]/-20[20]
15	6F/BM1-4C	47 < 2n>,XX,+9[20]
59	6F/BM1-4C	48 < 2n>,XX,+21,+22, 90 < 4n>,XXXX,-18,-20[20]/-21,-22[20]
61	6F/BM1-4C	47 < 2n>,XX,+X[20]/+9[20]

*4F2L-6C* episomal-induced system containing six reprogramming factors (*Oct4*, *Sox2*, *Klf4*, *c-Myc*, *SV40-LT*, and *Lin28*) and six compounds (PD, CHIR, NaB, Par, thi, and A83-01), *6F/BM1-4C* episomal-induced system containing six reprogramming factors (*Oct4*, *Glis1*, *Klf4*, *Sox2*, *L-Myc*, and miR-302cluster) and four compounds (PD, CHIR, NaB, and Par), [20] 20 metaphases, *<2n >* abnormal frequency ≥ 3 metaphases, *<4n >* abnormal frequency ≥ 15%

Reference to International System for Human Cytogenetic Nomenclature 2009

## Discussion

Reflecting their high efficiency and controllable cost, episomal plasmid-carried reprogramming factors are the most widely used approach for obtaining non-integrated iPSCs. Most episomal induction methods employ at least one oncogene or tumorigenic molecule, such as *c-Myc, SV40-LT*, *p53* short hairpin RNA (shRNA), and the p53 small molecule inhibitor cyclin pifithrin-α [10, 12–17], and iPSCs induced using previous methods cannot be used in clinical applications. Furthermore, induction methods that do not include tumorigenic factors are essential. In the present study, we constructed a low-risk 6F/BM1-4C reprogramming system, in which we eliminated the tumorigenic factors used in traditional episomal reprogramming systems, such as *c-Myc*, *SV40-LT*, and p53 inhibitor, and included *Oct4*, *Glis1*, *Klf4*, *Sox2*, *L-Myc*, the miR-302 cluster and four compounds, and then treated cells for no longer than 48 h to efficiently generate iPSCs from hUCs. This system also successfully converted hUCs from different sources into iPSCs and showed good reproducibility. Analyzing a large number of iPSCs by karyotype analysis, the 6F/BM1-4C-hiPSCs we generated exhibited fewer chromosome abnormalities compared with traditional 4F2L-6C-hiPSCs. In addition, expression of *Sirt1*, the NAD-dependent deacetylase necessary for maintaining iPSC genomic stability [37], in 6F/BM1-4C-iPSCs was high compared with iPSCs induced using the 4F2L-6C system, suggesting that 6F/BM1-4C-iPSC chromosome inheritance is more stable. Moreover, the presented method has a low cost, and the use of episomal plasmids makes this system suitable for clinical non-integrated iPSC preparation.

To obtain large-scale amounts of clinical-grade iPSCs, a reprogramming method with good reproducibility, non-tumorigenic reprogramming factors, and cost-effectiveness is needed; xeno-free components and a medium for primary somatic cell isolation to iPSC generation are also necessary. Besides, although the 6F/BM1-4C reprogramming system has relatively high reprogramming reproducibility, due to the heterogeneity of hUCs [18, 33, 34], it is difficult to accurately and separately perform multiplication culture in vitro to obtain a variety of different types of cells that meet the required number of experiments; hUCs from different donors and different batches of cells also show a wide range of induction efficiencies in the 6F/BM1-4C reprogramming system (Table 1). Therefore, except for a xeno-free induction reprogramming system, in the future the best reprogramming system should be screened for different types of hUCs or general suitability for a variety hUCs; in addition, the particular type of hUCs that is more easily reprogrammed or the particular type of hUCs that is more suitable for the 6F/BM1-4C system should be screened to find a more specific cultivation method for a particular type of hUCs, so that we can obtain a high-efficiency reprogramming system for screening high-quality clinical-grade iPSCs from a large number of iPSCs. Furthermore, when we used the xeno-free hESC E8 medium [38] to induce hUC reprogramming based on episomal vectors, we found it to be unsuitable after the addition of certain compounds, with deformed cells all dying (data not shown). Xeno-free extracellular matrices such as Vitronectin exhibit poor maintenance of iPSC self-renewal capacity [38]. Conversely, Laminin521 maintains iPSC self-renewal capacity, but it is extremely expensive [39] and thus is not suitable for large-scale production of clinical-grade iPSCs. Accordingly, the selection of appropriate cell culture materials remains essential for further clinical applications.

## Conclusion

We developed a safe method based on an episomal vector for inducing iPSCs from hUCs. This method does not involve the use of tumorigenic factors, such as *c-Myc*, *SV40-LT*, and p53 inhibitor. Karyotype analysis revealed that the chromosomal variation that occurred during iPSC generation in the present study was significantly low compared with the traditional method. Such low variability is critical for clinical applications of iPSCs.

## Additional files

Additional file 1: Figure S1. showing expression of factors from episomal vectors. **a** pE3.1 plasmid construction process chart (upper). Schematic representation of seven constructed episomal vectors. pEF1α *EF1α* promoter, pCMV CMV promoter (below). **b** Quantitative real-time PCR assay for *Oct4*, *Glis1*, *Klf4*, *Sox2*, *L-Myc*, linc-RoR, miR-367, miR-302a, miR-302b, miR-302c, and miR-302d. **c** Western blot assay for Oct4, Glis1, Klf4, Sox2, and L-Myc carried on episomal vectors. GAPDH was used as the loading control. (PDF 171 kb)

Additional file 2: Table S1. Presenting information for primers or functional fragments used in the present study. (XLSX 17 kb)

Additional file 3: Table S2. presenting plasmid combinations used for screening low-risk factors. (XLSX 9 kb)

Additional file 4: Table S3. presenting the method of karyotype analysis. (XLSX 10 kb)

Additional file 5: Figure S2. showing use of hUC16 cells to screen for low-risk factors for iPSC generation. **a** hUC16 morphology. **b** Strategy to screen low-risk factor combinations using hUC16 cells. **c** AP staining for iPSC generation using different factor combinations. **d** Numbers of AP-positive colonies. (PDF 137 kb)

Additional file 6: Figure S3. showing the effects of multiple compounds in the 6F combination system. **a** Strategy to optimize six-factor combinations using A83-01. **b** AP staining for iPSCs induced using A83-01. **c** Number of AP-positive colonies induced using A83-01. *P*(B) = 0.002. **d** Strategy to optimize six-factor combinations using Thiazovivin (thi). **e** AP staining of iPSCs induced using thi. **f** Number of AP-positive colonies induced using thi. *P*(D) = 0.000. **g** Strategy to optimize six-factor combinations using forskolin, PS48, and sc-79. **h** AP staining for iPSCs induced using forskolin, PS48, and sc-79. **i** Number of AP-positive colonies induced using forskolin, PS48, and sc-79. **j** Strategy to optimize six-factor combinations using DMOG and DZNEP. **k** AP staining for iPSCs induced using DMOG and DZNEP. **l** Number of AP-positive colonies induced using DMOG and DZNEP. *P*(C) = 0.041. **m** Strategy to optimize six-factor combination using Parnate in the early induction stage. **n** AP staining for iPSCs induced using Parnate in the early induction stage. Arrow indicates cell edge hemming. **o** Strategy to optimize six-factor combination treated with Parnate for a short time. **p** AP staining for iPSCs induced using Parnate for a short time. **q** Number of AP-positive colonies. *P*(C) = 0.04. Error bars indicate mean ± SD. *P < 0.05, **P < 0.01, ***P < 0.001. Scale bars, 100 μm. (PDF 227 kb)

Additional file 7: Figure S4. showing non-integrating analysis and karyotype assays of iPSCs induced with the 6F/BM1-4C system. **a** Non-integrating analysis of genomic DNA in iPSCs. Representative lanes: 1, H_2_O; 2, pE3.1-OG--KS and pE3.2-L-Myc--hmiR-302 cluster; 3, UC5; 4, UC5, pE3.1-OG--KS, and pE3.2-L-Myc--hmiR-302 cluster; 5, UC5iPSC1; 6, UC5iPSC2; 7, UC6; 8, UC6, pE3.1-OG--KS, and pE3.2-L-Myc--hmiR-302 cluster; 9, UC6iPSC1; 10, UC6iPSC2; 11, UC7; 12, UC7, pE3.1-OG--KS, and pE3.2-L-Myc--hmiR-302 cluster; 13, UC7iPSC1; 14, UC7iPSC2. OriP in lane 9 exhibited integration. **b** Karyotype analysis of iPSCs induced from several hUCs. (PDF 340 kb)

Additional file 8: Figure S5. showing pluripotent characterization of iPSCs induced using the 6F/BM1-4C system. **a** Flow cytometry for expression profiles of the hESC markers OCT4, SSEA4, Tra-1-60, and Tra-1-81. **b** Bisulfite sequencing assay for the methylation status of the *Oct4* and *Nanog* promoters in iPSCs. Color codes indicate the proportion of methylation. *y* axis shows individual CpGs analyzed. *x* axis shows different cells. **c** Immunofluorescence assay for expression profiles of hESC markers. **d** Quantitative real-time PCR assay for expression profiles of marker genes of the three germ layers. **e** Hematoxylin and eosin staining of sections of iPSC-generated teratomas. **f** Scatter plots comparing global gene expression patterns between HN4 hESCs and UC1 iPSCs and between UC2 cells and UC2 iPSCs. Highlighted are the pluripotency factors *Oct4*, *Sox2*, *Nanog*, and miR-302a. Error bars indicate mean ± SD. *P < 0.05, **P < 0.01, ***P < 0.001. Scale bars, 100 μm. (PDF 375 kb)

Additional file 9: Figure S6. showing morphology changes during iPSC generation using the 6F/BM1-4C system. **a** Morphology altered during iPSC generation using the 6F/BM1-4C system. **b** Schematic of episomal vectors used in the 4F2L-6C system. pEF1α *EF1α* promoter, pCMV CMV promoter. Scale bars, 100 μm. (PDF 158 kb)

## Abbreviations

iPSC: Induced pluripotent stem cell
hUC: Human urine-derived cell
hESC: Human embryonic stem cell
MCS: Multiple cloning site
AP: Alkaline phosphatase
EB: Embryoid body
PD: PD0325901
CHIR: CHIR99021
NaB: Sodium butyrate
Par: Parnate
thi: Thiazovivin
4C: PD, CHIR, NaB, and Par
4F2L-6C: episomal-induced system containing six reprogramming factors (*Oct4*, *Sox2*, *Klf4*, *c-Myc*, *SV40-LT*, and *Lin28*) and six compounds (PD, CHIR, NaB, Par, thi, and A83-01)
6C: PD, CHIR, NaB, Par, thi, and A83-01
6F/BM1-4C: episomal-induced system containing six reprogramming factors (*Oct4*, *Glis1*, *Klf4*, *Sox2*, *L-Myc*, and miR-302 cluster) and four compounds (PD, CHIR, NaB, and Par)
OSKM: *Oct4*, *Sox2*, *Klf4*, and *c-Myc*
6F: *Oct4*, *Glis1*, *Klf4*, *Sox2*, *L-Myc*, and the miR-302 cluster
4F2L: *Oct4*, *Sox2*, *Klf4*, *c-Myc*, *SV40-LT*, and *Lin28*
