# Supplementary figures and images for: Using low-risk factors to generate non-integrated human induced pluripotent stem cells from urine-derived cells
Source: Stem Cell Res Ther. 2017 Nov 2;8:245. doi: 10.1186/s13287-017-0698-8 (PMC5667457; doi:10.1186/s13287-017-0698-8)

Fig. S1

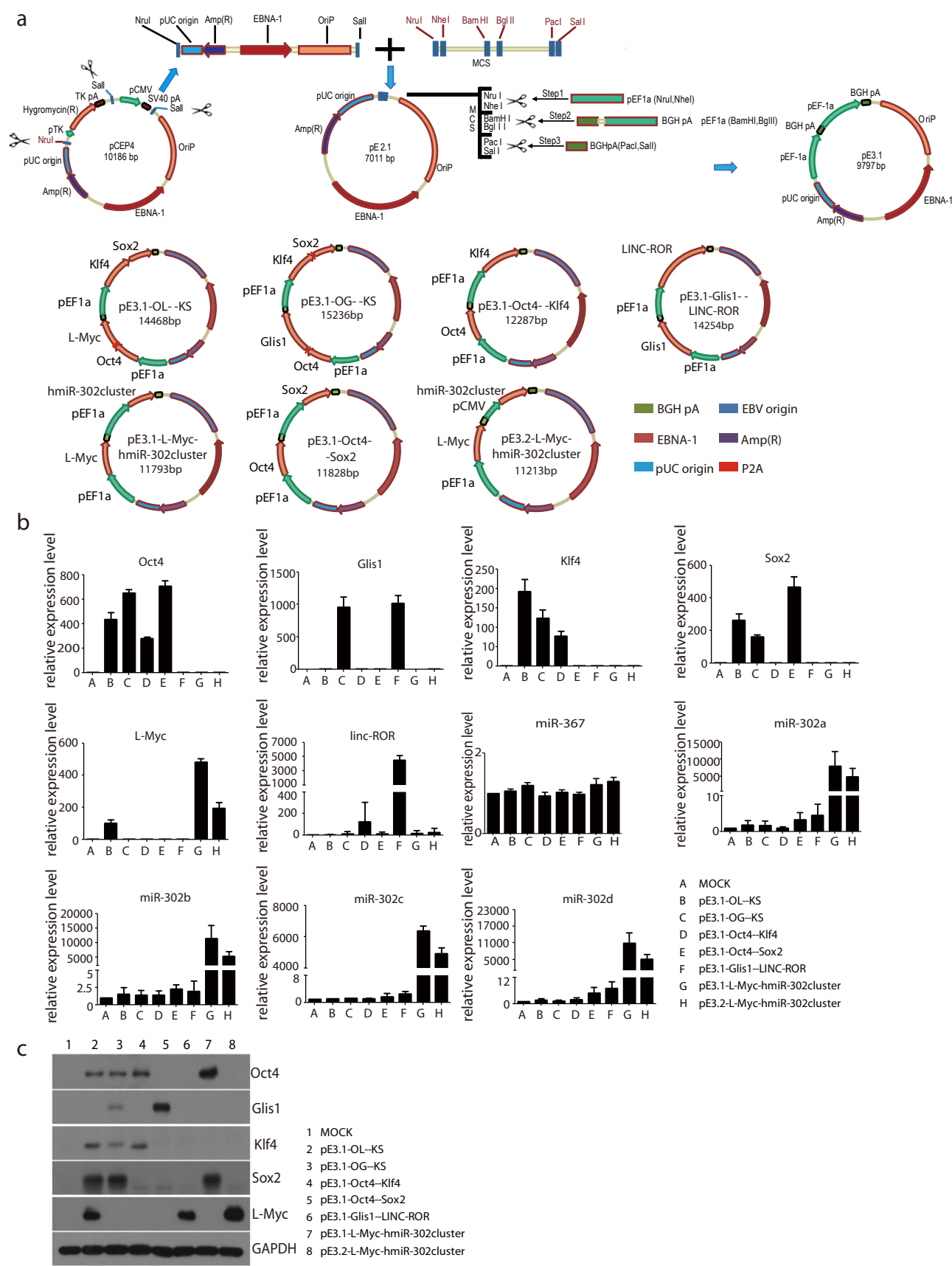

Supplement: Supplementary file 1 — showing expression of factors from episomal vectors. a pE3.1 plasmid construction process chart (upper). Schematic representation of seven constructed episomal vectors. pEF1α EF1α promoter, pCMV CMV promoter (below). b Quantitative real-time PCR assay for Oct4, Glis1, Klf4, Sox2, L-Myc, linc-RoR, miR-367, miR-302a, miR-302b, miR-302c, and miR-302d. c Western blot assay for Oct4, Glis1, Klf4, Sox2, and L-Myc carried on episomal vectors. GAPDH was used as the loading control. (PDF 171 kb) [file 13287_2017_698_MOESM1_ESM.pdf]

Fig. S2

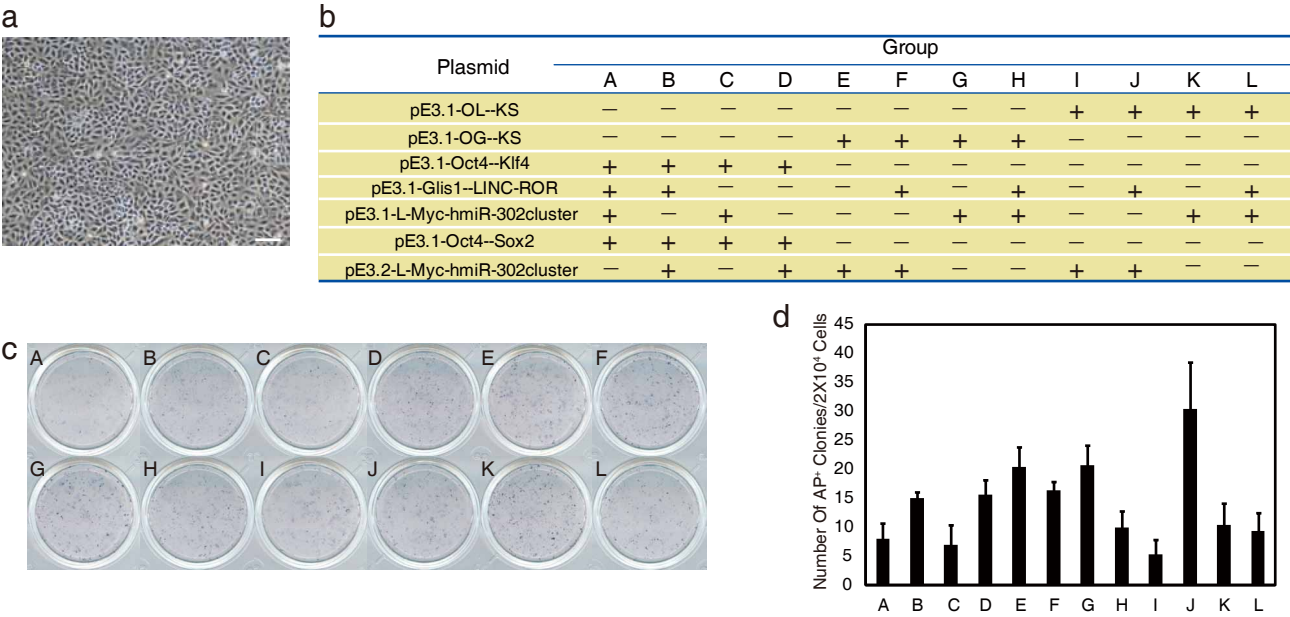

Supplement: Supplementary file 5 — showing use of hUC16 cells to screen for low-risk factors for iPSC generation. a hUC16 morphology. b Strategy to screen low-risk factor combinations using hUC16 cells. c AP staining for iPSC generation using different factor combinations. d Numbers of AP-positive colonies. (PDF 137 kb) [file 13287_2017_698_MOESM5_ESM.pdf]

Fig. S3

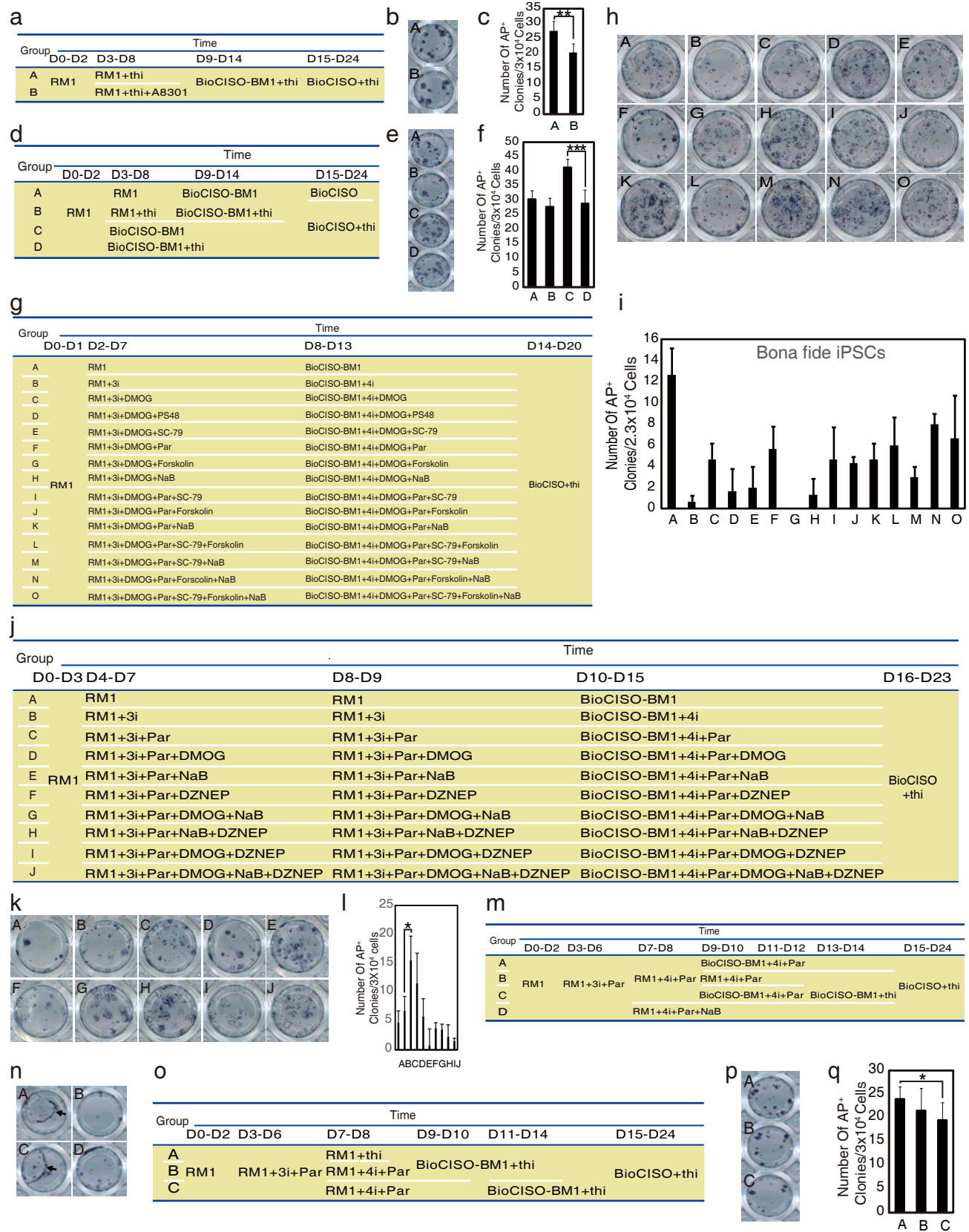

Supplement: Supplementary file 6 — showing the effects of multiple compounds in the 6F combination system. a Strategy to optimize six-factor combinations using A83-01. b AP staining for iPSCs induced using A83-01. c Number of AP-positive colonies induced using A83-01. P(B) = 0.002. d Strategy to optimize six-factor combinations using Thiazovivin (thi). e AP staining of iPSCs induced using thi. f Number of AP-positive colonies induced using thi. P(D) = 0.000. g Strategy to optimize six-factor combinations using forskolin, PS48, and sc-79. h AP staining for iPSCs induced using forskolin, PS48, and sc-79. i Number of AP-positive colonies induced using forskolin, PS48, and sc-79. j Strategy to optimize six-factor combinations using DMOG and DZNEP. k AP staining for iPSCs induced using DMOG and DZNEP. l Number of AP-positive colonies induced using DMOG and DZNEP. P(C) = 0.041. m Strategy to optimize six-factor combination using Parnate in the early induction stage. n AP staining for iPSCs induced using Parnate in the early induction stage. Arrow indicates cell edge hemming. o Strategy to optimize six-factor combination treated with Parnate for a short time. p AP staining for iPSCs induced using Parnate for a short time. q Number of AP-positive colonies. P(C) = 0.04. Error bars indicate mean ± SD. *P < 0.05, **P < 0.01, ***P < 0.001. Scale bars, 100 μm. (PDF 227 kb) [file 13287_2017_698_MOESM6_ESM.pdf]

Fig. S4

a

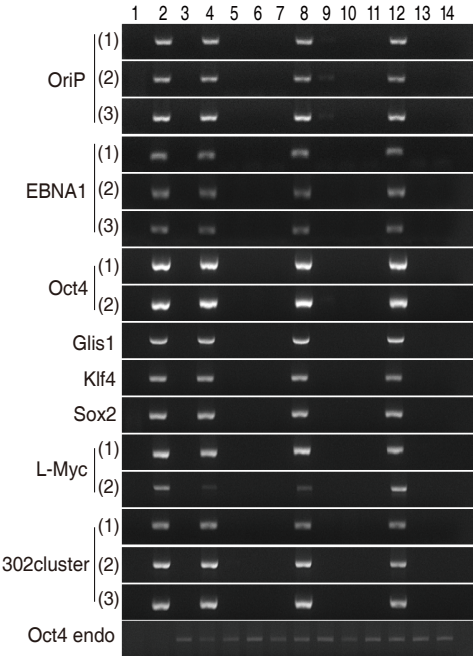

b

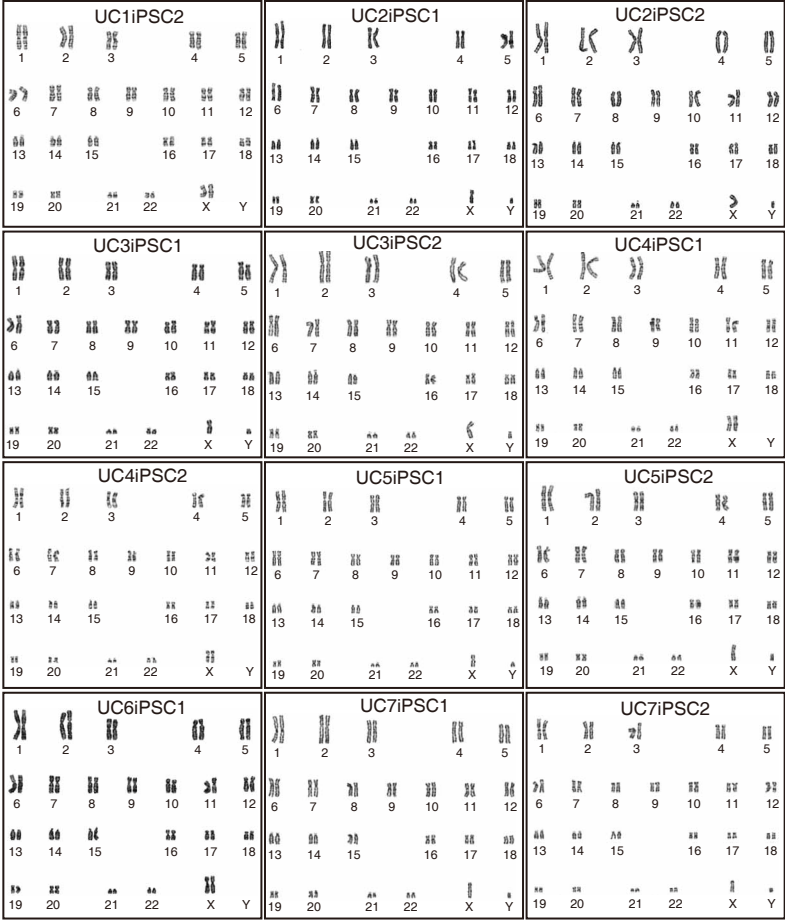

Supplement: Supplementary file 7 — showing non-integrating analysis and karyotype assays of iPSCs induced with the 6F/BM1-4C system. a Non-integrating analysis of genomic DNA in iPSCs. Representative lanes: 1, H2O; 2, pE3.1-OG--KS and pE3.2-L-Myc--hmiR-302 cluster; 3, UC5; 4, UC5, pE3.1-OG--KS, and pE3.2-L-Myc--hmiR-302 cluster; 5, UC5iPSC1; 6, UC5iPSC2; 7, UC6; 8, UC6, pE3.1-OG--KS, and pE3.2-L-Myc--hmiR-302 cluster; 9, UC6iPSC1; 10, UC6iPSC2; 11, UC7; 12, UC7, pE3.1-OG--KS, and pE3.2-L-Myc--hmiR-302 cluster; 13, UC7iPSC1; 14, UC7iPSC2. OriP in lane 9 exhibited integration. b Karyotype analysis of iPSCs induced from several hUCs. (PDF 340 kb) [file 13287_2017_698_MOESM7_ESM.pdf]

Fig.S5

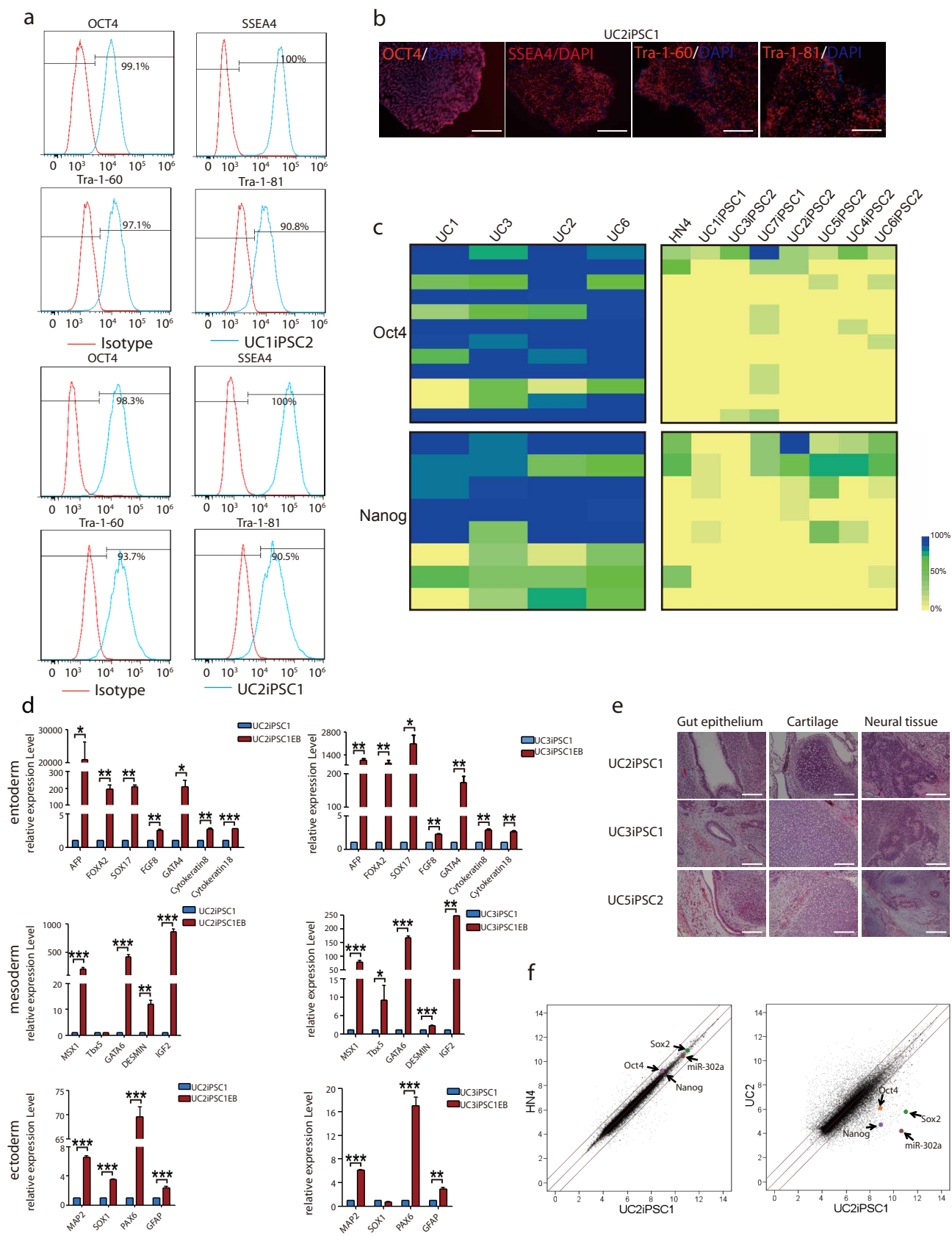

Supplement: Supplementary file 8 — showing pluripotent characterization of iPSCs induced using the 6F/BM1-4C system. a Flow cytometry for expression profiles of the hESC markers OCT4, SSEA4, Tra-1-60, and Tra-1-81. b Bisulfite sequencing assay for the methylation status of the Oct4 and Nanog promoters in iPSCs. Color codes indicate the proportion of methylation. y axis shows individual CpGs analyzed. x axis shows different cells. c Immunofluorescence assay for expression profiles of hESC markers. d Quantitative real-time PCR assay for expression profiles of marker genes of the three germ layers. e Hematoxylin and eosin staining of sections of iPSC-generated teratomas. f Scatter plots comparing global gene expression patterns between HN4 hESCs and UC1 iPSCs and between UC2 cells and UC2 iPSCs. Highlighted are the pluripotency factors Oct4, Sox2, Nanog, and miR-302a. Error bars indicate mean ± SD. *P < 0.05, **P < 0.01, ***P < 0.001. Scale bars, 100 μm. (PDF 375 kb) [file 13287_2017_698_MOESM8_ESM.pdf]

Fig. S6

a

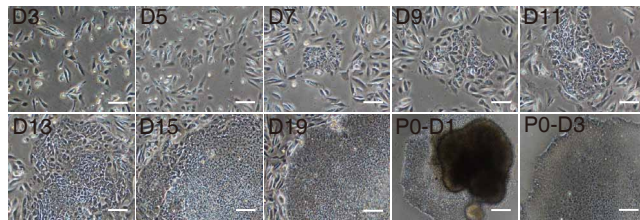

b

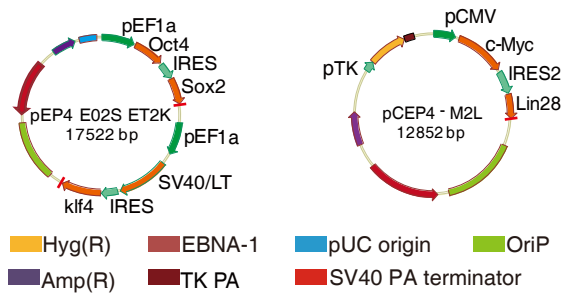

Supplement: Supplementary file 9 — showing morphology changes during iPSC generation using the 6F/BM1-4C system. a Morphology altered during iPSC generation using the 6F/BM1-4C system. b Schematic of episomal vectors used in the 4F2L-6C system. pEF1α EF1α promoter, pCMV CMV promoter. Scale bars, 100 μm. (PDF 158 kb) [file 13287_2017_698_MOESM9_ESM.pdf]
